# Factors affecting the sensitivity of human-derived esophageal carcinoma cell lines to 5-fluorouracil and cisplatin

**DOI:** 10.3892/ol.2012.1014

**Published:** 2012-11-05

**Authors:** TETSUYA MINEGAKI, KOHJI TAKARA, RYOHEI HAMAGUCHI, MASAYUKI TSUJIMOTO, KOHSHI NISHIGUCHI

**Affiliations:** 1 Department of Clinical Pharmacy, Faculty of Pharmaceutical Sciences, Kyoto Pharmaceutical University, Kyoto 607-8414;; 2 Department of Clinical Pharmaceutics, Faculty of Pharmaceutical Sciences, Himeji Dokkyo University, Himeji 670-8524, Japan

**Keywords:** esophageal carcinoma, cisplatin, 5-fluorouracil, drug sensitivity

## Abstract

Effective chemotherapy against esophageal carcinoma is considered achievable with a combination of 5-fluorouracil (5-FU) and cisplatin (CDDP). However, chemo-therapy remains ineffective in certain patients. The aim of this study was to clarify the factors which affect sensitivity to 5-FU and CDDP. The effects of factors known to influence sensitivity to 5-FU and CDDP, namely transporters, DNA repair enzymes and metabolic enzymes, were examined. mRNA levels of four transporters, SLC22A2, SLC23A2, ABCB1 and ABCC2, two DNA repair-related enzymes, Rad51 and MSH2, and one metabolic enzyme, dihydropyrimidine dehydrogenase (DPYD), showed a strong correlation (|r|>0.7) with IC_50_ values for 5-FU. In addition, the mRNA levels of ABCC2, MSH2 and DPYD showed a strong correlation (|r|>0.7) with the IC_50_ values for CDDP. Gimeracil, a DPYD inhibitor, enhanced the sensitivity of some cells to 5-FU but decreased the sensitivity of all the cells to CDDP. The inhibitory effects of ABCC2 with MK571 did not correspond to those observed in the correlation analysis. In conclusion, mRNA levels of SLC22A2, SLC23A2, ABCB1, ABCC2, Rad51, MSH2 and DPYD were confirmed to be strongly correlated with IC50 values for 5-FU, and mRNA levels of ABCC2, MSH2 and DPYD were confirmed to be strongly correlated with IC_50_ values for CDDP. In addition, the inhibition of DPYD appeared to affect the cytotoxicity of CDDP.

## Introduction

In Japan, one-third of all mortalities are cancer-related (1). The incidence of lung, colorectal and breast cancer is increasing in Japan as well as worldwide (1). Esophageal carcinoma has a lower incidence than other types of cancer, but 5-fluorouracil (5-FU) and cisplatin (CDDP)-based chemoradiotherapy results in moderately high response and survival rates relative to other types of cancer. In fact, the complete response and 5-year survival rates following 5-FU and CDDP-based chemoradiotherapy have been reported to be 58 and 29%, respectively, among Japanese esophageal carcinoma patients (2). However, chemotherapy remains ineffective in certain patients. Therefore, identifying the factors that affect sensitivity to 5-FU and CDDP is necessary for enhancing the clinical outcome of chemotherapy for esophageal carcinoma.

Certain factors affecting sensitivity to 5-FU or CDDP have previously been revealed, including the molecular mechanisms involved in the cellular kinetics and dynamics of 5-FU and CDDP. For example, overexpression of the ABC transporter superfamily C5 (ABCC5/MRP5) decreases cellular accumulation of 5-FU, resulting in resistance to 5-FU (3). In addition, dihydropyrimidine dehydrogenase (DPYD), a 5-FU metabolizing enzyme, has been correlated with clinical response to 5-FU-based chemotherapy among colon cancer patients (4,5). The cytotoxic effects of CDDP are also attenuated by ERCC1, a DNA repair-related enzyme associated with restoration of DNA damage induced by chemotherapeutic agents or UV rays (6–8). However, there is little information concerning whether the levels of these molecules are predictive of sensitivity to 5-FU or CDDP in esophageal carcinoma.

In the present study, sensitivity to 5-FU and CDDP and mRNA levels of 35 genes, including drug transporters, DNA repair enzymes and metabolic enzymes, were evaluated in 5 human esophageal carcinoma cell lines. Based on these findings, factors affecting the sensitivity of esophageal carcinoma cells to 5-FU and CDDP were examined.

## Materials and methods

### Chemicals

5-FU was obtained from Sigma-Aldrich Chemical Co. (St. Louis, MO, USA). CDDP was purchased from Wako Pure Chemical Industries, Ltd. (Osaka, Japan). Gimeracil and MK571 were purchased from Toronto Research Chemicals, Inc. (Toronto, ON, Canada) and Cayman Chemical Company (Ann Arbor, MI, USA), respectively. 2-(4-Iodophenyl)-5-(2,4-disulfophenyl)-2H-tetrazolium, monosodium salt (WST-1) and 1-methoxy-5-methylphenazinium methylsulfate were purchased from Dojindo Laboratories (Kumamoto, Japan).

### Cell culture

The human esophageal adenocarcinoma cell line OE33 was purchased from DS Pharma Biomedical Co., Ltd. (Osaka, Japan) and the squamous carcinoma cell lines KYSE30, KYSE70, KYSE140 and KYSE150 (9) were obtained from Health Science Research Resources Bank (Osaka, Japan). OE33 and the other cell lines were maintained in RPMI-1640 medium (Invitrogen Corp., Carlsbad, CA, USA) and Dulbecco’s modified Eagle’s medium (Invitrogen), respectively, supplemented with 10% heat-inactivated fetal bovine serum (lot no. 1335770 and 348777, Invitrogen). Cells were cultured in an atmosphere of 95% air and 5% CO_2_ at 37°C and subcultured every 3 or 4 days at a density of 1×10^6^ cells/25 cm^2^ culture flask. The number of passages for OE33, KYSE30, KYSE70, KYSE140 and KYSE150 cells was 15–25, 15–28, 15–26, 21–31 and 19–31, respectively.

### Growth rate of esophageal carcinoma cell lines

The growth rate of esophageal carcinoma cells was evaluated with a WST-1 assay utilizing succinate dehydrogenase activity. Cells were seeded onto a 96-well plate (Corning Inc., Corning, NY, USA) at a density of 5×10^3^ cells/well/100 *μ*l and cultured in an atmosphere of 95% air and 5% CO_2_ at 37°C. After 0, 6, 12, 18, 24, 36, 48, 72 and 96 h, the culture medium was exchanged for 110 *μ*l of medium containing WST-1 reagent solution (10 *μ*l WST-1 solution and 100 *μ*l culture medium), and 3 h later the absorbance was determined using a micro-plate reader at 450 nm with a reference wavelength of 620 nm (SpectraFluor™, Tecan Group Ltd., Männedorf, Switzerland). The doubling time for cell growth was calculated from the logarithmic phase of a growth curve (10) as follows: Doubling time = (t_1_ - t_0_) × log_10_2/(log_10_N_1_ - log_10_N_0_). N_0_ and N_1_ are the number of cells (% of day 0) at t_1_ and t_0_, respectively.

### Growth inhibitory activity assay

Cells were seeded onto 96-well plates (Corning Inc.) at a density of 5×10^3^ cells/well/100 *μ*l on day 0. After incubation for 24 h, the culture medium was exchanged for one containing 5-FU or CDDP at various concentrations (day 1). On day 4, a WST-1 assay was performed as described above.

The effects of gimeracil and MK571 on the growth inhibitory effects of 5-FU and CDDP were also evaluated by WST-1 assay. Cells were incubated for 24 h as described above and the culture medium was exchanged for one containing 5-FU or CDDP at various concentrations with or without gimeracil (100 *μ*M) or MK571 (50 *μ*M). Following incubation for 72 h at 37°C, the culture medium was replaced with a medium containing WST-1 and the absorbance was measured.

The 50% growth inhibitory concentrations (IC_50_) were calculated according to the sigmoid inhibitory effect model: E = E_max_ × [1 - C^γ^/(C^γ^ + IC_50_^γ^)], using the nonlinear least-squares fitting method (Solver, Microsoft^®^ Excel). E and E_max_ represent the surviving fraction (% of control) and its maximum, respectively. C and γ are the drug concentration in the medium and the sigmoidicity factor, respectively. Relative sensitivity was calculated as follows: Relative sensitivity = IC_50_ (without gimeracil or MK571)/IC_50_ (with gimeracil or MK571).

### Real-time reverse transcription (RT)-PCR

The mRNA expression levels were measured by real-time RT-PCR. Cells were seeded at a density of 2×10^6^ cells/60 mm culture dish and 48 h later, total RNA was extracted from the cells with a GenEluteTM Mammalian Total RNA Miniprep kit (Sigma-Aldrich). Total RNA (1 *μ*g) was used for RT with a PrimeScriptTM RT reagent kit (Takara Bio, Inc., Shiga, Japan) and a thermal cycler (i-Cycler, Bio-Rad Laboratories, Inc., Hercules, CA, USA). The RT reaction was conducted in 40 *μ*l reaction buffer at 37°C for 15 min and terminated by heating at 85°C for 5 sec followed by cooling at 4°C.

Real-time PCR was performed with a 7500 Real-time PCR system (Applied Biosystems, Carlsbad, CA, USA) and SYBR Premix Ex Taq™ (Takara Bio, Inc.). The primer sequences are shown in Table I. PCR was performed at 95°C for 10 sec, followed by 40 cycles of 95°C for 5 sec and 60°C for 34 sec. Dissociation was initiated at 95°C for 15 sec followed by 60°C for 1 min and 95°C for 15 sec. To compare the relative expression of target mRNA levels between the cell lines, the comparative Ct method was used, as previously described (10); β-actin (ACTB) was used as an internal standard. Samples were prepared in duplicate and three independent sample sets were analyzed.

### Statistical analyses

Data are shown as the mean ± standard deviation (SD). Comparisons between 2 and among 3 or more groups were performed with Student’s unpaired t-test and repeated one-way analysis of variance (ANOVA) followed by Scheffe’s F test, respectively. P<0.05 (two-tailed) was considered to indicate a statistically significant result. The correlation analysis was performed using Pearson’s correlation coefficient (r).

## Results

### Growth rates of esophageal carcinoma cell lines

Table II shows the cell growth doubling times for the 5 esophageal carcinoma cell lines. Doubling times for the cells varied from 20 to 25 h, revealing a significant difference between lines. KYSE30 cells (20.1±1.41 h) had the shortest doubling time and OE33 cells (25.0±0.90 h) the longest.

### Sensitivity of esophageal carcinoma cell lines to 5-FU and CDDP

The IC_50_ values for 5-FU were markedly different among the cell lines (0.524–30.2 *μ*M); the OE33 cells showed the highest sensitivity to 5-FU and the KYSE30 cells the lowest sensitivity (Table III). In the case of CDDP, the IC_50_ values were also substantially different among the cell lines (2.17–19.5 *μ*M). The rank order of sensitivity to CDDP was comparable to that for 5-FU.

### Correlation analysis of factors affecting drug sensitivity

The level of mRNA expression differed among the esophageal carcinoma cell lines (Table IV). The correlations between the IC_50_ values and the mRNA levels of the 35 different genes were analyzed (Table V). SLC22A3 mRNA was not detected in any cells, with the exception of the OE33 cell line. ABCC6 mRNA expression was not observed in KYSE30 and KYSE70 cells.

**Figure 1.**
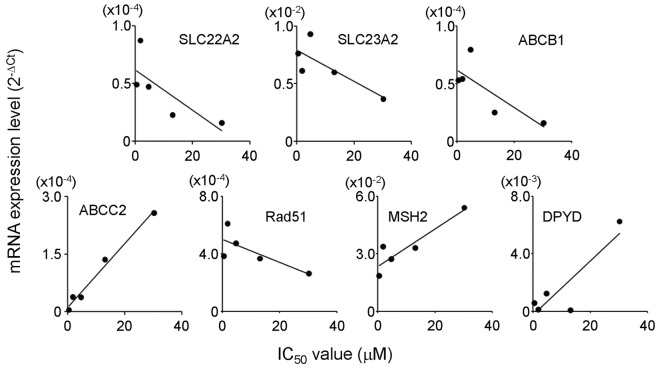
Correlation between IC_50_ values for 5-FU and mRNA expression levels in the esophageal carcinoma cell lines. The IC_50_ values for 5-FU were obtained from growth inhibition studies (Table III). The mRNA expression levels (2^−ΔCt^) in the cells were evaluated by real-time RT-PCR assay using SYBR^®^-Green. The threshold cycle (Ct) values were used to quantify the PCR product, and the relative expression level of the target gene was expressed as 2^−ΔCt^. The ΔCt was calculated by subtracting Ct (β-actin; as an internal standard) from Ct (target gene). 5-FU, 5-fluorouracil; RT, reverse transcription.

**Figure 2.**
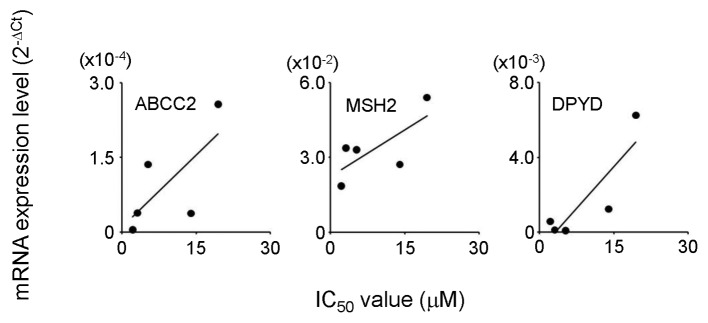
Correlation between the IC_50_ values for CDDP and mRNA expression levels in the esophageal carcinoma cell lines. The IC_50_ values for CDDP were obtained from growth inhibition studies (Table III). The mRNA expression levels (2^−ΔCt^) in the cells were evaluated by real-time RT-PCR assay using SYBR^®^-Green. The threshold cycle (Ct) values were used to quantify the PCR product, and the relative expression level of the target gene was expressed as 2^−ΔCt^. ΔCt was calculated by subtracting Ct (β-actin; as an internal standard) from Ct (target gene). CDDP, cisplatin; RT, reverse transcription.

The mRNA levels of SLC22A2, SLC23A2, ABCB1 and Rad51 showed a strong negative correlation (r<−0.7) with the IC_50_ values for 5-FU. ABCC2, MSH2 and DPYD were positively correlated with the IC_50_ values for 5-FU (r>0.7; Table V and Fig. 1). In the case of CDDP, a high positive correlation coefficient (r>0.7) was found between the IC_50_ values and ABCC2, MSH2 and DPYD mRNA expression (Table V and Fig. 2).

### Effects of gimeracil and MK571 on sensitivity of esophageal carcinoma cell lines to 5-FU and CDDP

The sensitivity of KYSE30 cells to 5-FU was enhanced by gimeracil, but in the other cell lines gimeracil had no observable effect (Table VI). In addition, gimeracil showed a tendency to decrease the sensitivity of all the cell lines to CDDP.

MK571 had no observable effect on the KYSE30, KYSE140 and KYSE150 cells (Table VII). However, the sensitivity of KYSE70 cells to 5-FU was substantially accelerated by the presence of MK571, and the sensitivity of OE33 cells to 5-FU was markedly decreased. However, MK571 showed a tendency to decrease sensitivity to CDDP, with the exception of the KYSE30 and KYSE150 cell lines.

## Discussion

Combination chemotherapy with 5-FU and CDDP is known to be effective against esophageal carcinoma. However, it remains ineffective in certain patients, and the causes for this have not been clarified. The aim of the present study was to examine the factors affecting the sensitivity of esophageal carcinoma cells to 5-FU and CDDP.

The sensitivity of the 5 different esophageal carcinoma cell lines to 5-FU and CDDP differed (Table III). OE33, an adenocarcinoma cell line, showed a high sensitivity to 5-FU and CDDP, whereas the squamous cell carcinoma KYSE30 cells showed low sensitivity to 5-FU and CDDP. In addition, OE33 cells had the longest doubling time (an index of cell growth) of all the cell lines and KYSE30 cells the shortest (Table II), resulting in a trend for lower sensitivity to chemo-therapeutic agents among cells with higher growth activity. These findings suggest that sensitivity to 5-FU and CDDP was influenced by the growth activity of cells, although cytotoxic agents such as 5-FU and CDDP are known to be more toxic in cells with higher growth activity. In order to resolve this discrepancy, further studies concerning the correlation between cell growth and sensitivity to 5-FU or CDDP should be performed.

The correlations between sensitivity to 5-FU and CDDP and the mRNA levels of the 35 genes were then examined. The levels of target mRNA expression differed among the cell lines (Table IV). The mRNA levels of ABCC2, MSH2 and DPYD were positively correlated with the IC_50_ values of 5-FU (r>0.7; Fig. 1 and Table V). By contrast, a negative correlation between the IC_50_ values of 5-FU and the mRNA levels of SLC22A2, SLC23A2, ABCB1 and Rad51 was observed. In the light of the biological roles of these genes, the negative correlation between SLC22A2 and SLC23A2 mRNA expression and sensitivity was considered to be noteworthy. SLC22A2 encodes an organic cation transporter which is responsible for cell uptake of various drugs, including CDDP (11,12). A colon carcinoma cell line exhibiting resistance to 5-FU has been reported to show lower expression of SLC23A2 mRNA than its parent cells (13). ABCC2, MSH2 and DPYD are known to act in detoxifying mechanisms; they are an efflux transporter, DNA repair-related protein and metabolic enzyme, respectively. Although ABCB1 is a known efflux transporter that contributes to drug resistance, the cytotoxicity of 5-FU was not influenced by the expression of ABCB1 (14). In addition, the overexpression of DNA-repair related proteins, including Rad51, has been reported to contribute to resistance to DNA damaging agents (15). Although the present findings showing a negative correlation between IC_50_ values and ABCB1 and Rad51 mRNA expression levels conflict with previous findings, they may indicate that ABCB1 and Rad51 have no significant impact on sensitivity.

In the case of CDDP, a positive correlation (r>0.7) between the IC_50_ values and the mRNA levels of ABCC2, MSH2 and DPYD was identified. The findings for ABCC2 and MSH2 are supported by their functions; the export of CDDP from cells (16) and repair of DNA damaged by CDDP (17), respectively (Table V and Fig. 2). In addition, proliferating cell nuclear antigen-normalized mRNA expression of DPYD has previously been reported to be associated with sensitivity to CDDP in lung cancer tissues (18). Although the correlation between CDDP and DPYD has not been investigated in detail, these previous results may support the present findings. The mRNA levels of ABCC2, MSH2 and DPYD correlated well with sensitivity to both 5-FU and CDDP, suggesting that these are potent predictive factors for 5-FU and CDDP-based chemotherapy in esophageal carcinoma patients.

Finally, the roles of ABCC2 and DPYD in sensitivity to 5-FU and CDDP were examined, since the knock-down of MSH2 in SW460 and HeLa cells has been reported to have no influence on sensitivity to 5-FU (19). In the present study, 100 *μ*M gimeracil, which showed sufficient inhibition of DPYD (20), enhanced 5-FU sensitivity in the KYSE30 cell line (Table VI), which had the highest level of DPYD mRNA expression of all the cell lines tested (Table IV). The present findings support those of Ando *et al*(21); that is, DPYD was a predictor of sensitivity to 5-FU. Apart from the correlation analysis, gimeracil decreased sensitivity to CDDP in all cell lines (Table VI), implying that DPYD activity may be required for the cytotoxic effect of CDDP. Further investigations are required to resolve this contradiction. The concomitant administration of 50 *μ*M MK571, a representative ABCC2 inhibitor (22), was found to decrease the sensitivity of OE33 and KYSE150 cells to 5-FU. In addition, the growth inhibitory activity of CDDP was decreased in KYSE30 and KYSE150 cell lines (Table VII). These findings conflict with the function of ABCC2 function as an efflux transporter, and further investigations are required to clarify this situation.

In conclusion, the mRNA levels of SLC22A2, SLC23A2, ABCB1, ABCC2, Rad51, MSH2 and DPYD were confirmed to be strongly correlated with the IC_50_ values for 5-FU, and those of ABCC2, MSH2 and DPYD were also confirmed to be strongly correlated with the IC_50_ values for CDDP. These genes have the potential to affect the sensitivity to 5-FU and CDDP. In addition, the inhibition of DPYD was suggested to affect the cytotoxicity of CDDP. These findings provide useful information for improving the clinical outcome of chemotherapy against esophageal carcinoma.

## Figures and Tables

**Table I.** Sequences of oligonucleotide primers designed for real-time PCR.

Function and gene	Forward (5′–3′)	Reverse (5′–3′)	Reference
ACTB	TCATGAAGTGTGACGTGGACATC	TGCATCCTGTCGGCAATG	10
Transport			
SLC22A1	TCTTCCATCGTCACTGAGTTCAAC	AGAAGCCCGCATTCAAACAG	10
SLC22A2	TCTACTCTGCCCTGGTTGAATTC	ATGCAGCCCAAGGGTAACG	10
SLC22A3	TAGCCCCATTTCTGCTCTTTC	AGATGGATGCCAGGATACCAA	10
SLC23A2	TCTTTGTGCTTGGATTTTCGAT	ACGTTCAACACTTGATCGATTC	23
SLC31A1	ACAAGTCAGCATTCGCTACAATTC	TTGCAGGAGGTGAGGAAAGC	9
ABCB1	TTCCTTCACCCAGGCAATG	ATGAGTTTATGTGCCACCAAGTAG	^a^
ABCC1	CAGTGACCTCTGGTCCTTAAACAA	TTGGCGCATTCCTTCTTCC	24
ABCC2	ACTTGTGACATCGGTAGCATGGA	AAGAGGCAGTTTGTGAGGGATGA	^a^
ABCC3	GTCCGCAGAATGGACTTGAT	TCACCACTTGGGGATCATTT	25
ABCC4	GCTCAGGTTGCCTATGTGCT	CGGTTACATTTCCTCCTCCA	25
ABCC5	CGAAGGGTTGTGTGGATCTT	GTTTCACCATGAAGGCTGGT	^a^
ABCC6	TGTCGCTCTTTGGAAAATCC	AGGAACACTGCGAAGCTCAT	25
ABCG2	TGACGGTGAGAGAAAACTTAC	TGCCACTTTATCCAGACCT	26
ATP7A	AGATACTGGGACACTGGAGAAA	AGGTCATCCCTTCCACTTTCA	10
ATP7B	TGATTTATAACCTGGTTGGGATACC	ATGAGAGCACCACAGACACAGA	10
DNA repair			
ERCC1	TACAAGGCCTATGAGCAGAAACCA	TCTCTTGATGCGGCGATGAG	^a^
ERCC2	CTGGAGGTGACCAAACTCATCTA	CCTGCTTCTCATAGAAGTTGAGC	27
ERCC3	TATCCCAGGACACACAGGAAAT	TCACCTTGAAGCTATAACCTTGA	^a^
XPA	TGCGGCGAGCAGTAAGAAG	TCATGGCCACACATAGTACAAGTC	^a^
Rad51	TGGGAACTGCAACTCATCTGG	GCGCTCCTCTCTCCAGCAG	28
BRCA1	ACAGCTGTGTGGTGCTTCTGTG	CATTGTCCTCTGTCCAGGCATC	29
BRCA2	TGAAGAGCAGTTAAGAGCCTTGAA	ACGGTTGTGACATCCCTTGATAAA	^a^
HMGB1	CAAGCGAACAGCAGGGTTAG	CAGATTGAGTCATTTGCTCCTCTTA	^a^
HMGB2	TGAACATCGCCCAAAGATCA	TCAGACCACATTTCACCCAATT	^a^
MLH1	GATTACCCCTTCTGATTGACA	ACTGAGGCTTTCAAAACA	30
MSH2	CAGTATATTGGAGAATCGCA	AGGGCATTTGTTTCACC	30
PMS2	AGTCAGCGTGCAGCAGTTATT	GACCATTTTGGCATACTCCTTCT	^a^
RPP25	AGAATGGTGGACAGTGGGATT	TACTTCAGGTGCTCTTCGTGAATG	^a^
Metabolism			
GSTP1	CTGCGCATGCTGCTGGCAGATC	TTGGACTGGTACAGGGTGAGGTC	31
GCLC	GGCAAGATACCTTTATGACCAGTT	TGCAGCACTCAAAGCCATAA	32
GCLM	TGACTGCATTTGCTAAACAATTTGA	CGTGCGCTTGAATGTCAGG	33
TYSM	GCCTCGGTGTGCCTTTCA	CCCGTGATGTGCGCAAT	34
DPYD	AATGATTCGAAGAGCTTTTGAAGC	GTTCCCCGGATGATTCTGG	35
UMPS	TAGTGTTTTGGAAACTGTTGAGGTT	CTTGCCTCCCTGCTCTCTGT	36
MTHFR	CGGGTTAATTACCACCTTGTCAA	GCATTCGGCTGCAGTTCA	36

a Primer sequences were designed using Primer Express^®^ software. ACTB, β-actin.

**Table II.** Doubling times of esophageal carcinoma cell lines.

Cell line	Doubling time, mean ± SD (h)
OE33	25.0±0.90
KYSE30	20.1±1.41
KYSE70	21.8±0.51
KYSE140	23.3±1.07
KYSE150	20.6±0.53

n=6.

**Table III.** IC_50_ values for 5-FU and CDDP in esophageal carcinoma cell lines.

	IC_50_ value, mean ± SD (*μ*M)	
Cell line	5-FU	CDDP
OE33	0.524±0.08	2.17±0.33
KYSE30	30.2±8.29	19.5±3.67
KYSE70	13.1±13.3	5.27±0.36
KYSE140	1.88±0.38	3.09±0.67
KYSE150	4.75±1.46	14.0±1.02

n=4. 5-FU, 5-fluorouracil; CDDP, cisplatin.

**Table IV.** Expression levels of mRNA in esophageal carcinoma cell lines.

	Expression ratio, mean ± SD (2^−ΔCt^×10^−4^)				
Function and gene	OE33	KYSE30	KYSE70	KYSE140	KYSE150
Transport					
SLC22A1	0.11±0.03	0.07±0.02	0.01±0.003	0.03±0.02	0.12±0.07
SLC22A2	0.49 ±0.15	0.16±0.03	0.23±0.03	0.87±0.45	0.47±0.36
SLC22A3	36.3±9.24	ND	ND	ND	ND
SLC23A2	76.1±13.8	36.6±6.39	59.8±4.66	61.1±43.8	92.9±64.0
SLC31A1	125±26.6	131±10.2	179±23.4	252±135	244±147
ABCB1	0.53±0.14	0.16±0.03	0.25±0.06	0.54±0.20	0.79±0.59
ABCC1	74.7±11.3	37.0±3.83	246±32.9	123±75.6	67.8±36.6
ABCC2	0.05±0.01	2.57±0.89	1.36±0.07	0.38±0.16	0.38±0.23
ABCC3	80.5±15.1	10.2±2.64	60.7±8.62	40.6±23.3	123±86.7
ABCC4	26.1±2.17	28.9±1.95	52.1±5.53	189±105	92.5±51.3
ABCC5	9.06±1.30	62.14±17.0	65.38±8.60	22.92±10.6	26.76±19.2
ABCC6	1.79±0.12	ND	ND	0.05±0.05	0.04±0.04
ABCG2	6.25±1.29	4.64±0.21	1.66±0.34	2.47±0.69	33.1±18.3
ATP7A	8.66±1.27	7.99±0.70	4.68±1.20	6.09±3.61	15.4±7.98
ATP7B	1.91±0.25	1.89±0.73	2.21±0.47	5.72±4.77	3.37±2.69
DNA repair					
ERCC1	219±66.1	143±35.8	96.3±13.2	241±131	296±175
ERCC2	43.9±4.57	35.1±8.01	21.0±2.52	47.9±22.4	52.2±22.6
ERCC3	82.3±11.5	79.3±19.4	57.4±6.31	134±97.9	185±104
XPA	91.4±16.0	107±16.7	193±14.8	461±290	283±164
Rad51	3.84±1.03	2.64±0.37	3.67±1.09	6.09±3.07	4.72±1.75
BRCA1	90.5±15.6	61.1±2.46	65.3±4.56	188±116	151±78.8
BRCA2	110±20.4	111±3.99	43.5±3.74	261±165	204±108
HMGB1	35.2±7.29	35.9±1.84	51.4±3.98	79.9±47.8	37.6±19.1
HMGB2	509±87.3	1340±150	1343±129	1980±947	1367±679
MLH1	27.5±4.55	21.3±1.61	23.9±1.83	28.2±18.4	70.9±36.8
MSH2	185±39.8	540±38.6	331±23.5	338±154	272±114
PMS2	25.3±3.22	34.2±4.24	77.1±12.8	123±81.6	67.0±42.8
RPP25	27.5±4.35	6.32±0.99	0.13±0.03	74.9±59.9	0.74±0.47
Metabolism					
GSTP1	2444 ±425	2926±644	3421±380	5784±3549	7249±3978
GCLC	5.21±0.51	3.87±1.15	45.0±4.30	10.8±4.41	9.05±5.39
GCLM	8.38±2.61	31.34±4.30	75.1±10.8	33.0±15.4	32.1±22.2
TYMS	54.0±11.6	163±3.10	2511±136	81.8±32.9	215.6±102
DPYD	5.81±2.03	62.4±6.50	0.82±0.29	1.23±0.87	12.4±9.82
UMPS	85.6±17.4	69.0±3.85	162±20.6	183±108	165±73.2
MTHFR	5.78±1.85	10.0±2.39	18.6±2.72	25.6±23.2	23.7±15.3

ΔCt = Ct (target gene) - Ct (β-actin). ND, not detected; n=3.

**Table V.** Pearson’s correlation coefficient between IC_50_ values for 5-FU or CDDP and mRNA expression level.

	Pearson’s correlation coefficient (r)	
Function and gene	5-FU	CDDP
Transport		
SLC22A1	−0.189	0.333
SLC22A2	−0.764	−0.574
SLC22A3	ND	ND
SLC23A2	−0.790	−0.302
SLC31A1	−0.477	−0.132
ABCB1	−0.788	−0.215
ABCC1	−0.150	−0.530
ABCC2	0.992^b^	0.706
ABCC3	−0.659	−0.179
ABCC4	−0.470	−0.315
ABCC5	0.573	0.234
ABCC6	ND	ND
ABCG2	−0.244	0.398
ATP7A	−0.199	0.451
ATP7B	−0.485	−0.314
DNA repair		
ERCC1	−0.638	−0.041
ERCC2	−0.533	0.019
ERCC3	−0.439	0.187
XPA	−0.463	−0.284
Rad51	−0.756	−0.523
BRCA1	−0.653	−0.274
BRCA2	−0.455	−0.049
HMGB1	−0.341	−0.507
HMGB2	0.010	0.125
MLH1	−0.369	0.269
MSH2	0.913^a^	0.719
PMS2	−0.363	−0.365
RPP25	−0.486	−0.561
Metabolism		
GSTP1	−0.401	0.121
GCLC	0.032	−0.321
GCLM	0.287	−0.011
TYMS	0.163	−0.211
DPYD	0.881	0.863
UMPS	−0.522	−0.379
MTHFR	−0.319	−0.074

ND, not detected.

a P<0.05 and

b P<0.01 significant correlations between IC_50_ values and mRNA expression levels. 5-FU, 5-fluorouracil; CDDP, cisplatin.

**Table VI.** Relative sensitivity of the esophageal carcinoma cell lines to 5-FU or CDDP with or without gimeracil.

	Relative sensitivity, mean ± SD (fold)	
Cell line	5-FU	CDDP
OE33	1.10±0.37	0.579±0.06
KYSE30	2.30±0.13	0.710±0.03
KYSE70	1.16±0.19	0.687±0.05
KYSE140	0.989±0.15	0.691±0.10
KYSE150	1.19±0.16	0.788±0.25

Relative sensitivity, the ratio of IC_50_ value for 5-FU or CDDP without gimeracil to those with gimeracil (n=4). Gimeracil, 100 *μ*M. 5-FU, 5-fluorouracil; CDDP, cisplatin.

**Table VII.** Relative sensitivity of the esophageal carcinoma cell lines to 5-FU or CDDP with or without MK571.

	Relative sensitivity, mean ± SD (fold)	
Cell line	5-FU	CDDP
OE33	0.0680±0.01	0.852±0.28
KYSE30	0.961±0.06	0.974±0.09
KYSE70	2.36±1.36	0.617±0.06
KYSE140	0.813±0.16	0.803±0.04
KYSE150	0.731±0.11	1.08±0.26

Relative sensitivity, the ratio of IC_50_ values for 5-FU or CDDP without MK571 to those with MK571 (n=4). MK571, 50 *μ*M. 5-FU, 5-fluorouracil; CDDP, cisplatin.
